# Molecular and Pharmacological Characterization of the Interaction between Human Geranylgeranyltransferase Type I and Ras-Related Protein Rap1B

**DOI:** 10.3390/ijms22052501

**Published:** 2021-03-02

**Authors:** Sonja Hinz, Dominik Jung, Dorota Hauert, Hagen S. Bachmann

**Affiliations:** 1 Center for Biomedical Education and Research (ZBAF), Faculty of Health, Institute of Pharmacology and Toxicology, School of Medicine, University of Witten/Herdecke, 58453 Witten, Germany; Sonja.Hinz@uni-wh.de (S.H.); dominik.jung@uni-wh.de (D.J.); dorota.hauert@uni-wh.de (D.H.); 2 PharmaCenter Bonn, Pharmaceutical Institute, Pharmaceutical & Medicinal Chemistry, Rheinische Friedrich-Wilhelms-Universität Bonn, An der Immenburg 4, 53121 Bonn, Germany

**Keywords:** NanoBiT assay, geranylgeranyltransferase type I, Rap1B, GGTI-298, Adenosine A_2B_- and A_2A_-receptor

## Abstract

Geranylgeranyltransferase type-I (GGTase-I) represents an important drug target since it contributes to the function of many proteins that are involved in tumor development and metastasis. This led to the development of GGTase-I inhibitors as anti-cancer drugs blocking the protein function and membrane association of e.g., Rap subfamilies that are involved in cell differentiation and cell growth. In the present study, we developed a new NanoBiT assay to monitor the interaction of human GGTase-I and its substrate Rap1B. Different Rap1B prenylation-deficient mutants (C181G, C181S, and ΔCQLL) were designed and investigated for their interaction with GGTase-I. While the Rap1B mutants C181G and C181S still exhibited interaction with human GGTase-I, mutant ΔCQLL, lacking the entire CAAX motif (defined by a cysteine residue, two aliphatic residues, and the C-terminal residue), showed reduced interaction. Moreover, a specific, peptidomimetic and competitive CAAX inhibitor was able to block the interaction of Rap1B with GGTase-I. Furthermore, activation of both Gα_s_-coupled human adenosine receptors, A_2A_ (A_2A_AR) and A_2B_ (A_2B_AR), increased the interaction between GGTase-I and Rap1B, probably representing a way to modulate prenylation and function of Rap1B. Thus, A_2A_AR and A_2B_AR antagonists might be promising candidates for therapeutic intervention for different types of cancer that overexpress Rap1B. Finally, the NanoBiT assay provides a tool to investigate the pharmacology of GGTase-I inhibitors.

## 1. Introduction

Post-translational modifications are important processes to expand and enhance protein functions in response to complex external stimuli. One example is the prenylation of proteins that are involved in biological regulation in eukaryotic cells [1]. Geranylgeranyltransferase-I (GGTase-I) and farnesyltransferase (FTase) are prenyltransferases that catalyze the transfer of a 20-carbon or a 15-carbon isoprenoid from geranylgeranyl diphosphate (GGPP) or farnesyl diphosphate (FPP), respectively, to proteins with a *C*-terminal CAAX motif—the CAAX motif is defined by a cysteine residue (C), two aliphatic residues (AA) and the *C*-terminal residue (X, determines enzyme prevalence; in the case of GGTase-I X refers mostly to leucine) that contribute to substrate specificity [2,3]. Both enzymes are functional as cytosolic heterodimers formed by an α- and a β-subunit. The α-subunits of FTase and GGTase-I are identical (hereafter referred to as FTase α), whereas the β-subunits are different and harbor the active site. In different studies it was demonstrated that truncated rat (ΔN29) and yeast FTase α-subunits are able to form homodimers [4]. In contrast, investigations conducted with full-length human FTase α-subunits showed no indication of homodimer formation by human FTase α [5]. Geranylgeranyltransferase type II (GGTase-II or RabGGTase) is more specialized and modifies only G-proteins from the Rab subfamily. Additionally, a recently identified geranylgeranyltransferase type III (GGTase-III) consists of an orphan prenyltransferase α-subunit and the catalytic β-subunit of GGTase-II [6,7].

One important substrate of GGTase-I is the Ras-like small GTPase Rap1, which has two isoforms Rap1A and Rap1B that differ only in a few amino acids [8]. The Rap1 protein has different cellular functions depending on its isoform and subcellular localization. For example, high Rap1B expression has been detected in different squamous cell carcinoma cell lines [9]. Many studies have indicated that Rap1B is required for cell survival and migration in human cancer cell models [10] and is targeted by several miRNAs [11,12,13].

The fact that prenylation is usually—depending on the cell-type—required for the oncogenic activity of small GTPases led to the development of GGTase-I inhibitors as potential anti-cancer drugs [14,15,16,17,18,19]. Recently, FGTI-2734—a Ras *C*-terminal mimetic dual farnesyltransferase and geranylgeranyltransferase inhibitor—inhibited membrane localization of *K*-Ras in human pancreatic, lung and colon cancer cells. Moreover, the compound induced apoptosis and inhibited the growth of mutant *K*-Ras-dependent human tumors in mice [20]. In a phase 1 study with the peptidomimetic small molecule inhibitor GGTI-2418 in patients with advanced solid tumors, the compound was safe and tolerable at all tested dose levels but was rapidly eliminated and could not achieve optimal GGTase-I inhibition [21]. Furthermore, GGTase inhibitors are postulated to have therapeutic potential in many other diseases, including multiple sclerosis, arteriosclerosis, viral infection, as well as osteoporosis and were developed as antifungals and antiparasitics [17,22]. The principal mechanism of the competitive, peptidomimetic CAAX GGTase inhibitors is to block the interaction of GGTase with their substrates, leading to lack of prenylation and therefore to mislocalization. However, many of the structurally diverse GGTase inhibitors were never characterized in detail, and their exact mode of action is unknown. Additionally, half maximal inhibitory concentration (IC_50)_ data for the same compound are often in disagreement when using different cell lines and assay systems.

In the present study, we developed a new NanoBiT assay to characterize the interaction of human GGTase-I and its substrate Rap1B in real-time. This assay system uses a split luciferase system (NanoLuc) consisting of an optimized small (1.3 kDa peptide, SmBiT) and a large (18 kDa polypeptide, LgBiT) NanoLuc subunit. Moreover, compared to other methods that are suitable to measure protein–protein interactions, such as most of the bimolecular fluorescence complementation systems (BiFC) [23], the interaction is reversible, and temporally dynamic protein interactions can be monitored in real-time [24]. Additionally, the NanoBiT technology was already evaluated successfully for many different drug targets, such as G protein-coupled receptors (GPCRs) and enzymes [25,26,27,28,29,30,31]. 

Here, the assay was also used to investigate the blocking effect of the specific GGTase-I inhibitor *N*-[4-[2(*R*)-amino-3-mercaptopropyl]amino-2-(1-naphthalenyl)benzoyl]-*L*-leucine methyl ester trifluoroacetate salt (GGTI-298) and the effect of A_2A_AR (adenosine A_2A_ receptor) and A_2B_AR (adenosine A_2B_ receptor) signaling on human GGTase-I and Rap1B interaction. The A_2A_AR and A_2B_AR are G protein-coupled receptors that are co-expressed on many different cell types, e.g., on cells of the immune system. Both receptors play important roles in inflammation and have recently become major drug targets in immuno-oncology [32]. A_2B_ARs are highly expressed on different cancer cells, promoting cancer cell proliferation and angiogenesis, and A_2B_AR antagonists have been suggested for cancer therapy [33,34,35,36,37,38]. Moreover, it has been shown that A_2A_AR and A_2B_AR are able to form—dependent on their expression levels in different cell lines—heterodimers with an altered pharmacology compared to monomers [39,40]. Both receptors activate adenylate cyclase via Gα_s_-proteins, and the A_2B_AR can additionally couple to calcium mobilization, mainly via G_q_-proteins in different cell lines [41,42,43,44]. The A_2A_ARs are activated by low nanomolar adenosine concentrations, whereas A_2B_ARs require higher concentrations, which are only reached under hypoxic or inflammatory conditions. Ntantie et al. have identified a signaling pathway by which activation of the A_2B_AR with the non-selective adenosine receptor (AR) agonist 5′-*N*-ethylcarboxamidoadenosine (NECA) causes protein kinase A (PKA) to phosphorylate the polybasic region (PBR) of newly synthesized Rap1B [45]. This inhibits the interaction of Rap1B with the chaperone protein SmgGDS-607 and suppresses Rap1B prenylation (Supplementary Figure S1). That in turn leads to reduced Rap1B trafficking to the plasma membrane and to increased cell-scattering and tumor metastasis [45]. Reduced Rap1B prenylation was detected in different cancer cell lines and tumors [46].

In the present study, we further investigated whether the activation of both Gα_s_-coupled A_2A_AR and A_2B_AR have an effect on human GGTase-I and Rap1B interaction and thereby potentially modulate the prenylation of Rap1B. Additionally, different Rap1B mutants (C181S, C181G, ΔCQLL) were evaluated to characterize the human GGTase-I and Rap1B interaction on the molecular level. 

## 2. Results

### 2.1. Development of a NanoBiT Assay to Monitor the Interaction of Human GGTase-I and Rap1B

The NanoBiT system provides a new platform to measure protein–protein interactions in living cells and in real-time. Here, we developed a new NanoBiT assay to monitor the interaction of the human GGTase-type-I and its substrate Rap1B. We used human embryonic kidney cells (HEK293) for our experiments because it was previously shown by qRT-PCR, Western blots, and pharmacologically evaluated, functional cyclic adenosine monophosphate (cAMP) accumulation assays that HEK293 cells express endogenous A_2B_ARs and A_2A_ARs (Supplementary Figure S5) [41,44,47,48,49]. The A_2A_AR selective agonist CGS-21680 was able to increase cAMP accumulation in HEK293-A_2B_ cells with an ED_50_-value of 15.2 µM [44]. Moreover, the HEK293 cells utilized for this study were additionally stably transfected with human A_2B_AR [43] (Supplementary Figure S6) and co-transfected with combinations of SmBiT and LgBiT plasmid constructs. 

An overview of all NanoLuc fusion constructs used in this study is given in Supplementary Figure S2. The *N*-terminal orientation of the NanoBiT tags on Rap1B was chosen because the *C*-terminus of the Rap1B protein carries the CAAX motif, which is important for the recognition and interaction with the active site of the GGTase-I-β-subunit. Moreover, in previous studies it was shown that a HA-tag (hemagglutinin) at the *N*-terminus of Rap1B does not modify its subcellular localization or its ability to be phosphorylated by PKA in vivo [50]. Additionally, the SmBiT tag was used for the human Rap1B wild-type (WT) protein as well as for the Rap1B prenylation-deficient mutants C181G, C181S, and ΔCQLL, considering their relatively small size of ~ 21 kDa. The LgBiT was attached to the *N*-terminus of the human GGTase-I-β-subunit. For the structurally similar yeast GGTase-I, it was demonstrated that an *N*-terminal GS dipeptide or a Flag-tag at the β-subunit does not influence the function of the enzyme [51,52]. Furthermore, LgBiT and SmBiT tags were attached to the *N*-terminus of FTase α-subunit for control experiments. For initial experiments, the SmBiT-FTase α was co-transfected with LgBiT-GGTase-I-β-subunit as a heteromeric positive control, and the combination of human LgBiT-FTase α and SmBiT-FTase α served as a negative control [5]. Additionally, a non-interacting fusion protein (HaloTag^®^-SmBiT) was co-transfected in combination with LgBiT-GGTase-I-β-subunit as a second negative control. Moreover, LgBiT and SmBiT tags were attached to the *C*-terminus of A_2A_AR, which were co-transfected for A_2A_AR-homodimer control experiments [40,53].

A total of nine fusion proteins were generated and connected with a flexible glycine/serine linker sequence. Transfected plasmid amounts of 50 ng/well or 100 ng/well were evaluated, and first experiments were conducted with cells transfected solely with SmBiT or LgBiT plasmids, respectively. As expected, none of the mono-transfected cells (50 ng/well) showed any significant luminescence signal over a time period of 60 min (Supplementary Figure S3A,B).

**Figure 1:**
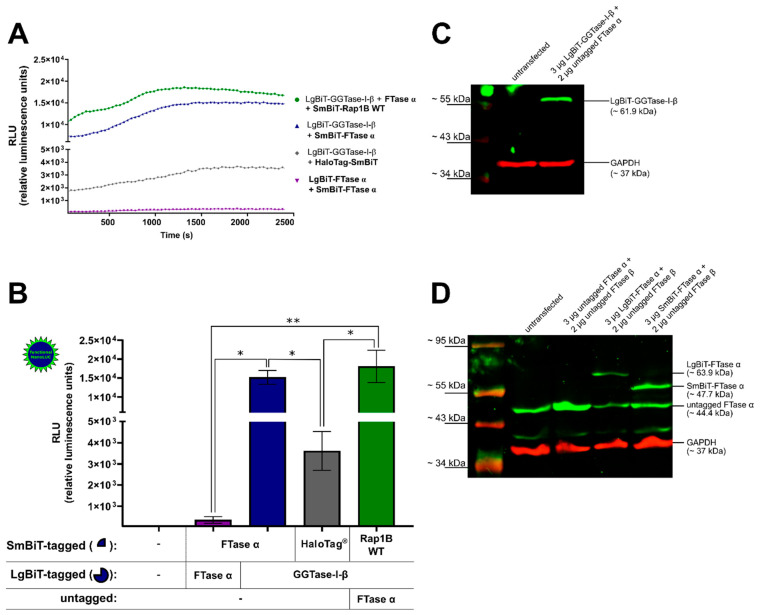
Development of a NanoBiT assay to measure the interaction of geranylgeranyltransferase type-I (GGTase-I) and Rap1B. Human embryonic kidney cells (HEK293) cells stably expressing the adenosine A_2B_ receptor (A_2B_AR) were transiently co-transfected with combinations of plasmids encoding LgBiT or SmBiT attached to the *N*-terminus of farnesyltransferase (FTase) α, GGTase-I-β, and Rap1B wild-type (WT) (each 50 ng DNA/well). The untagged FTase α pcDNA3.1 (−) plasmid was co-transfected (100 ng DNA/well) in combination with LgBiT-GGTase-I-β to form the functional heteromeric enzyme, which was then able to interact with SmBiT-Rap1B WT. The co-transfected combinations LgBiT-FTase α/SmBiT-FTase α and LgBiT-GGTase-I-β/HaloTag^®^-SmBiT (50 ng DNA/well) were used as negative controls, and the combination LgBiT-GGTase-I-β/SmBiT-FTase α served as a positive control. Additionally, for the Western blot analysis, the untagged FTase β pcDNA3.1 (+) was co-transfected in combination with untagged FTase α pcDNA3.1 (−), LgBiT-FTase α, and SmBiT-FTase α to form the functional heteromeric enzyme. (**A**) Real-time kinetic measurement of complex formation using the NanoBiT assay. Twenty-four hours after transfection, the NanoGlo live-cell substrate was added, and the association was immediately monitored at 25 °C over a time period of 42 min with a luminescence reading taken every 30 s. (**B**) After a time period of ~ 900–1835 s, the maxima of complex formation were reached (dependent on which plasmid combinations were used, Figure 1A), and the relative luminescence units (RLU) were plotted. Significant differences for the combinations LgBiT-GGTase-I-β/SmBiT-FTase α and LgBiT-GGTase-I-β/FTase α/SmBiT-Rap1B WT over the negative controls LgBiT-FTase α/SmBiT-FTase α and LgBiT-GGTase-I-β/HaloTag^®^-SmBiT were demonstrated (* *p* < 0.05, ** *p* < 0.01). (**C**) Representative Western blot analysis of LgBiT-GGTase-I-β expression in HEK293 cells stably expressing the A_2B_AR. A specific monoclonal first NanoLuc/LgBiT antibody and an infrared second antibody were used to detect the NanoLuc fusion protein LgBiT-GGTase-I-β (green, ~ 61.9 kDa). A specific first monoclonal glycerinaldehyd-3-phosphat-dehydrogenase (GAPDH) antibody and infrared second antibody were used to detect GAPDH (red, ~ 37 kDa) as a loading control. Two independent Western blots were performed. (**D**) Representative Western blot of untagged FTase α, LgBiT-FTase α, and SmBiT-FTase α expression in HEK293 cells stably expressing the A_2B_AR. A specific first anti-FTase α antibody and infrared second antibody were used to detect untagged FTase *α* (green, ~ 44.4 kDa), LgBiT-FTase α (green, ~ 63.9 kDa), and SmBiT-FTase α (green, ~ 47.7 kDa). The GAPDH antibodies were used as described above. Two independent Western blots were performed.

The co-transfected combination of LgBiT-GGTase-I-β-subunit (50 ng/well) and SmBiT-FTase α-subunit (50 ng/well), as well as the co-transfected combination of LgBiT-GGTase-I-β-subunit (50 ng/well), untagged FTase α-subunit (100 ng/well), and SmBiT-Rap1B WT (50 ng/well) were able to generate a robust luminescence signal, which was stable over a time frame of 42 min (Figure 1A). The untagged FTase α-subunit was co-transfected to form the heteromeric and functional GGTase-I. A slightly diminished luminescence signal was observed for the interaction of LgBiT-GGTase-I-β-subunit/FTase α-subunit/SmBiT-Rap1B WT compared to the LgBiT-GGTase-I-β/SmBiT-FTase-α-subunit heteromer (Figure 1A). Furthermore, co-transfection of LgBiT-GGTase-I-β-subunit (50 ng/well) with a non-interacting protein, HaloTag^®^-SmBiT (50 ng/well), did only yield a slight increase in luminescence. The signal was approximately 5-fold and significantly lower than the heteromeric positive control LgBiT-GGTase-I β-subunit/SmBiT-FTase α-subunit and the interaction between LgBiT-GGTase-I-β-subunit/FTase α-subunit/SmBiT-Rap1B WT (* *p* < 0.05) (Figure 1B). Moreover, co-transfection of SmBiT-FTase α-subunit (50 ng/well) and LgBiT-FTase α-subunit (50 ng/well) showed an approximately 50-fold lower luminescence signal compared to the heteromeric positive control LgBiT-GGTase-I β-subunit/SmBiT-FTase α-subunit (* *p* < 0.05) and the interaction between LgBiT-GGTase-I-β-subunit/FTase α-subunit/SmBiT-Rap1B WT (** *p* < 0.01) (Figure 1B). The expression of LgBiT-GGTase-I-β-subunit (~61.9 kDa) (Figure 1C), untagged FTase α-subunit (~44.4 kDa), LgBiT-FTase α-subunit (~63.9 kDa), and SmBiT-FTase α-subunit (~47.7 kDa) was confirmed by Western blots (Figure 1D). As the blots show, untransfected HEK293 cells expressed untagged FTase α, which represents the α-subunit of GGTase-I, endogenously (Figure 1D). The respective band intensified upon overexpression of untagged FTase α and remained in cells transfected with LgBiT-FTase α and SmBiT-FTase α (Figure 1D). 

### 2.2. Effect of the Competitive CAAX Peptidomimetic GGTase-I Inhibitor N-[4-[2(R)-amino-3-mercaptopropyl]amino-2-(1-naphthalenylbenzoyl]-L-leucine methyl ester trifluoroacetate salt (GGTI-298) on the Interaction of GGTase-I and Rap1B and the Formation of A_2A_AR Homodimers

**Figure 2:**
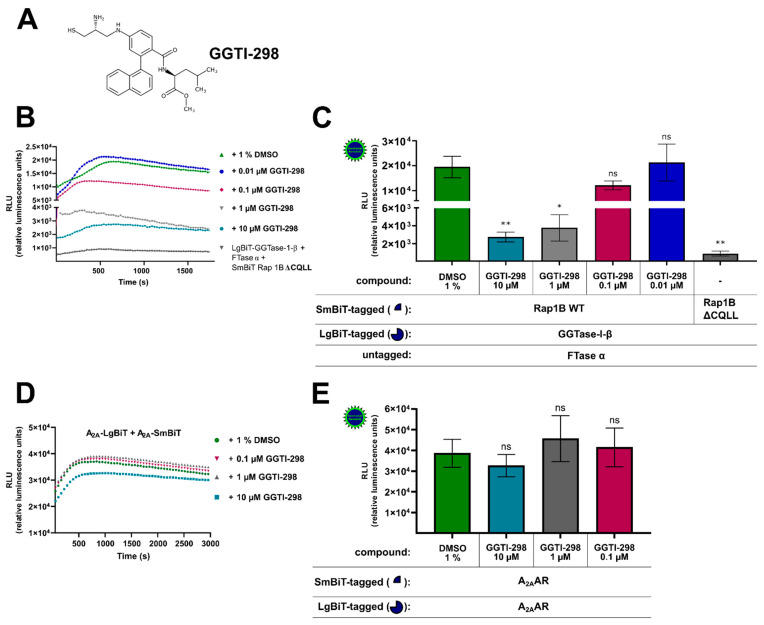
Effect of the cell-permeable, competitive CAAX (defined by a cysteine residue, two aliphatic residues, and the *C*-terminal residue) peptidomimetic GGTase-I inhibitor GGTI-298 on the interaction of GGTase-I and Rap1B and on A_2A_AR-homodimers. HEK293 cells stably expressing the adenosine A_2B_ receptor (A_2B_AR) were transiently co-transfected as described in Figure 1. From the Rap1B mutant, ΔCQLL 50 ng DNA/well was used. Additionally, for a control of the specificity of GGTI-298, HEK293 cells were transiently transfected with combinations of plasmids encoding LgBiT and SmBiT attached to the *C*-terminus of the human A_2A_AR (each 100 ng DNA/well). (**A**) Structure of the cell-permeable, competitive CAAX peptidomimetic GGTase-I inhibitor GGTI-298. (**B**) Twenty-four hours after transfection, the cells were pre-treated for 30 min with the selective GGTase-I inhibitor GGTI-298 in different concentrations (10 µM, 1 µM, 0.1 µM, 0.01 µM) or with 1% DMSO at 37 °C in OptiMEM medium. Then the NanoGlo live-cell substrate was added and the association was immediately monitored at 25 °C over a time period of 30 min with a luminescence reading taken every 30 s. (**C**) After a time period of ~ 400–700 s the maxima of complex formation were reached (dependent on which GTI-298 concentration was used, see (**B**), and the relative luminescence units (RLU) were plotted. Significant differences were observed between the untreated LgBiT-GGTase-I-β/FTase α/SmBiT-Rap1B WT complex and the GGTI-298 inhibitor (10 µM, 1 µM) treated Lg-BiT-GGTase-I-β/FTase α/SmBiT-Rap1B complex (* *p* < 0.05, ** *p* < 0.01). (**D**) After 24 h the A_2A_AR transfected HEK293 cells were pre-treated for 30 min with the selective GGTase-I inhibitor GGTI-298 in different concentrations (10 µM, 1 µM, 0.1 µM) or with 1% DMSO at 37 °C in OptiMEM medium. Then the NanoGlo live-cell substrate was added, and the association was immediately monitored at 25 °C over a time period of 50 min, with a luminescence reading taken every 30 s. (**E**) After a time period of ~ 1005 s, the maxima of A_2A_AR-homodimer formation were reached, and the relative luminescence units (RLU) were plotted. No significant differences were observed between the untreated A_2A_-LgBiT/A_2A_-SmBiT homodimer and the GGTI-298 inhibitor (10 µM, 1 µM, 0.1 µM) treated A_2A_-LgBiT/A_2A_-SmBiT homodimer (ns, not significant).

To further confirm the specificity of the assay, we tested whether the competitive and selective peptidomimetic CAAX motif inhibitor GGTI-298 (Figure 2A) was able to block the protein–protein interaction between Rap1B and the β-subunit of GGTase-I. GGTI-298 was shown to exhibit antitumor activity and inhibit the processing of geranylgeranylated Rap1A with an IC_50_-value of 3 µM [54,55]. In HEK293 cells stably expressing the A_2B_AR and co-transfected with LgBiT-GGTase-I-β-subunit/FTase α-subunit/SmBiT-Rap1B WT, GGTI-298 concentration dependently reduced the generated luminescence signal compared to cells that were only treated with 1% of dimethyl sulfoxide (DMSO) (Figure 2B,C). The GGTI-298-treated complex formation of LgBiT-GGTase-β-subunit/FTase α-subunit/SmBiT-Rap1B WT showed similar kinetic properties as the untreated (1% DMSO) complex formation of LgBiT-GGTase-I-β-subunit/FTase-α-subunit/SmBiT-Rap1B WT (Figure 2B). After a time period of ~ 400–700 s, the maxima of complex formation were reached (dependent on which GGTI-298 concentration was used, Figure 2B), and statistically significant differences of the inhibitor-treated complex (10 µM, 1 µM) compared to the untreated complex were observed (* *p* < 0.05, ** *p* < 0.01) (Figure 2C). Because of assay variability (transient transfection and therefore variations in construct expression/complex formation, different sample arrangements on the 96-well plate followed by pipetting the substrate manually), we could not quantify the exact time point of the maxima for each treated/untreated complex formation. We determined always the highest luminescence value for each assay/complex formation and defined it as maximum. Therefore, we conclude that, for example, the 1 µM GGTI-298 treated complex formation (maxima after ~ 400 s) exhibited similar kinetic properties compared to 10 µM GGTI-298 treated complex formation (maxima after ~ 700 s) (Figure 2B).

We considered the possibility that the decrease in luminescence with the GGTI-298 inhibitor was not the result of a specific competition versus Rap1B but rather a result of inhibitory effects on the reconstituted NanoLuc luciferase or of cytotoxicity. To investigate this possibility, we performed the same GGTI-298 inhibitor treatment with the well described A_2A_AR-homodimer [53]. As before, HEK293 cells were co-transfected with A_2A_-LgBiT/A_2A_-SmBiT and pre-treated for 30 min with different concentrations of GGTI-298 inhibitor (10 µM, 1 µM, 0.1 µM) or with 1% DMSO (Figure 2D,E). The GGTI-298 inhibitor treated A_2A_AR-homodimer showed similar kinetic properties as the untreated (1% DMSO) A_2A_AR-homodimer (Figure 2D). No significant differences of the GGTI-298 inhibitor treated A_2A_AR-homodimer (10 µM, 1 µM, 0.1 µM) compared to the untreated A_2A_AR-homodimer were observed (ns, not significant) (Figure 2E). Thus, we conclude that the observed GGTI-298 inhibitor effect was specific and blocked the interaction between GGTase-I and its substrate Rap1B.

### 2.3. Interaction of GGTase-I with Prenylation-Deficient Rap1B Mutants C181G, C181S, and ΔCQLL

Having established that the new NanoBiT assay is suitable to measure the interaction of human GGTase-I and Rap1B, we were interested in studying the interaction of human GGTase-I with prenylation-deficient Rap1B mutants. Rap1B contains a *C*-terminal hypervariable region, which consists of a polybasic region (PBR) and the CAAX motif. The CAAX motif contains the cysteine that is the site of prenylation and is important for the coordination of the Zn^2+^ ion in the active site of the enzyme [56]. Crystal structures are known of several complexes formed by the rat GGTase-I and different substrates [3,7], but no models or crystal structures are published for human GGTase-I and Rap1B complexes. We therefore further evaluated the effect of prenylation-deficient Rap1B mutants on the direct binding of human GGTase-I, which has not yet been characterized. For that, the cysteine in the CAAX motif was replaced by serine or glycine to generate the non-prenylateable Rap1B mutants C181S and C181G. Moreover, a complete CAAX motif deletion mutant, ΔCQLL, was generated. 

**Figure 3:**
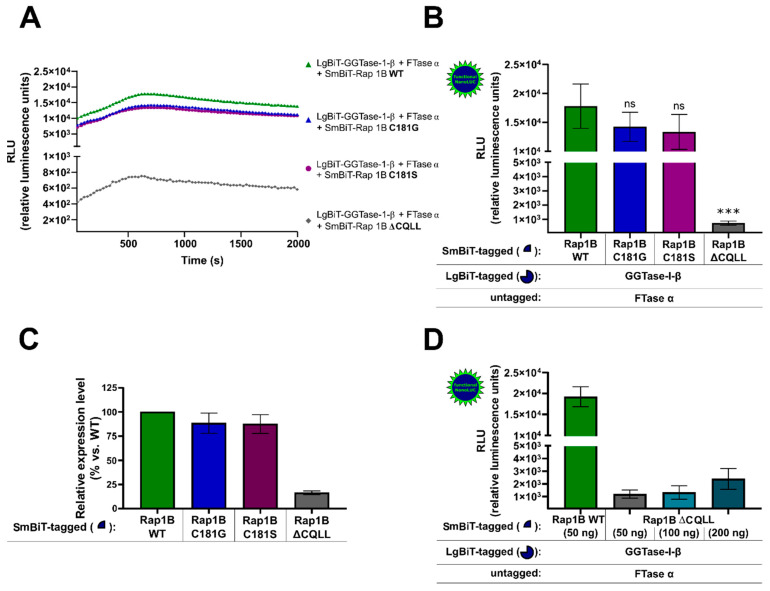
NanoBiT complementation assay to measure the interaction of GGTase-I and the Ras-related protein Rap1B mutants C181G, C181S, ΔCQLL. HEK293 cells stably expressing the A_2B_AR were transiently co-transfected as described in Figure 1. From the Rap1B mutants C181G, C181S, 50 ng DNA/well and from ΔCQLL 50–200 ng DNA/well were used. (**A**) Real-time kinetic measurement of heteromer formation using the NanoBiT assay. Twenty-four hours after transfection, the NanoGlo live-cell substrate was added, and the association was immediately monitored at 25 °C over a time period of 42 min with a luminescence reading taken every 30 s. (**B**) After a time period of ~ 700 s, the maxima of complex formation were reached, and the relative luminescence units (RLU) were plotted. No significant differences between LgBiT-GGTase-I-β/FTase α/SmBiT-Rap1B WT and the LgBiT-GGTase-I-β/FTase α/SmBiT-Rap1B mutants C181G, C181S were observed (ns, not significant). Significant differences were observed between LgBiT-GGTase-I-β/FTase α/SmBiT-Rap1BWT and the LgBiT-GGTase-I-β/FTase α/SmBiT-Rap1B mutant that was missing the CAAX motif (ΔCQLL) (*** *p* < 0.001). (**C**) Densitometric quantification of SmBiT-Rap1B WT, SmBiT-Rap1B C181G, SmBiT-Rap1B C181S, and SmBiT-Rap1B ΔCQLL expression in HEK293 cells. Protein levels are represented as ratio values quantified from protein bands of each sample versus GAPDH compared to SmBiT-Rap1B WT expression (set as 100% expression). Three independent Western blots were performed. (**D**) Increasing plasmid amounts of SmBiT-Rap1B ΔCQLL (50–200 ng DNA/well) were transiently co-transfected in HEK293 cells stably expressing the A_2B_AR. After the maxima of complex formation were reached, the relative luminescence units (RLU) were plotted.

HEK293 cells stably expressing the A_2B_AR were transiently co-transfected with either SmBiT-Rap1B WT and LgBiT-GGTase-I-β-subunit/FTase α-subunit or with the mutated SmBiT-Rap1Bs that were prenylation-deficient (C181G, C181S, ΔCQLL) and LgBiT-GGTase-I-β-subunit/FTase α-subunit. The kinetic properties of complex formations of Rap1B mutants C181G, C181S, and ΔCQLL were similar to those when using Rap1B WT (Figure 3A). The maxima of complex formation were reached after a time period of ~ 700 s (Figure 3A). The Rap1B mutants C181G and C181S showed a slight decrease but no significant differences in the luminescence signal compared to Rap1B WT (Figure 3B). In contrast, the luminescence signal of LgBiT-GGTase-I-β-subunit/FTase α-subunit/SmBiT-Rap1B ΔCQLL was approximately 20-fold lower compared to the interaction of LgBiT-GGTase-β-subunit/FTase α-subunit/SmBiT-Rap1B WT (*** *p* < 0.001) (Figure 3B). To exclude that the low luminescence signal of LgBiT-GGTase-I-β-subunit/FTase α-subunit/SmBiT-Rap1B ΔCQLL was due to a lack of expression, we performed Western blots and densitometric quantification of the protein bands (Figure 3C). The expression of SmBiT-Rap1B WT and SmBiT-Rap1B mutants C181G and C181S was comparable, as shown by immunoblotting (Figure 3C, Supplementary Figure S4), but the expression of SmBiT-Rap1B ΔCQLL was approximately 5-fold lower compared to the SmBiT-Rap1B WT (Figure 3C, Supplementary Figure S4). Increasing transfected plasmid amounts of SmBiT-Rap1B ΔCQLL only marginal increased the luminescence signals. The 4-fold transfected plasmid amount of SmBiT-Rap1B ΔCQLL (200 ng DNA/well) only increased the luminescence signal ~ 2-fold (Figure 3D). The luminescence signal of LgBiT-GGTase-I-β-subunit/FTase α-subunit/SmBiT-Rap1B ΔCQLL (200 ng DNA/well) was still approximately 10-fold lower compared to the luminescence signal generated by LgBiT-GGTase-I-β-subunit/FTase-α-subunit/SmBiT-Rap1B WT (50 ng DNA/well) (Figure 3D).

### 2.4. Effect of A_2B_AR Agonists and Antagonists on the Interaction of GGTase-I and Rap1B WT

Earlier studies showed that the treatment of cells with compounds that elevate intracellular cAMP (cyclic adenosine monophosphate) levels results in PKA-dependent Rap1B phosphorylation [50,57]. For example, forskolin treatment resulted in an increase of Rap1B phosphorylation and in an increase in the bound GTP/GDP ratio compared to the basal ratio [50]. In following studies, Wilson et al. showed that the activation of Gα_s_-coupled β-adrenergic receptors phosphorylates Rap1B and inhibits its prenylation. This led to disturbed membrane localization and promoted the metastatic phenotype in breast cancer cells [58]. The same group showed that activation of A_2B_AR with the non-selective AR agonist NECA phosphorylates newly synthesized Rap1B and inhibits its interaction with the chaperone protein SmgGDS-607, leading to a decreased Rap1B prenylation and signaling at the cell membrane. This in turn led to reduced cell–cell contact and promoted tumor metastasis [45,46,59]. However, the direct binding of Rap1B to human GGTase-I was never characterized in detail nor was the pharmacological effect of specific and non-specific A_2A_AR and A_2B_AR agonists and antagonists on this protein–protein interaction. 

**Figure 4:**
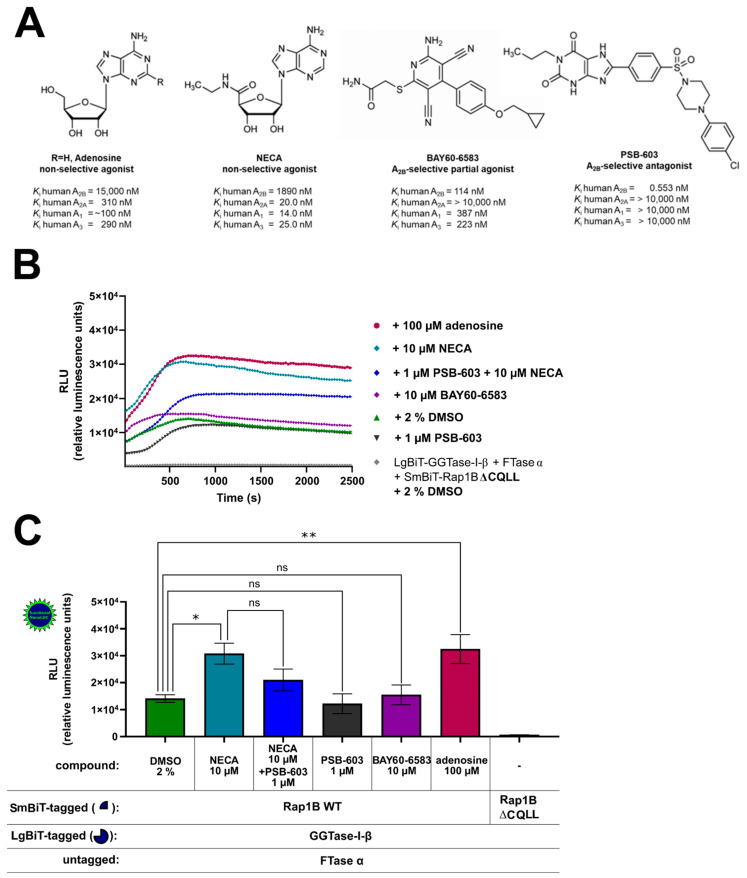
Effect of A_2B_AR agonists and antagonists on the interaction of GGTase-I and Rap1B WT. HEK293 cells stably expressing the A_2B_AR were transiently transfected as described above. (**A**) Selected non-selective AR agonists (adenosine, 5′-*N*-ethylcarboxamidoadenosine (NECA)), A_2B_AR-selective partial agonist [2-({6-amino-3,5-dicyano-4-[4-(cyclopropylmethoxy)phenyl]pyridin-2-yl}sulfanyl)acetamide] (BAY60-6583), A_2B_AR-selective antagonist (8-(4-[4-(4-chlorophenyl)piperazine-1-sulfonyl]phenyl)-1-propylxanthine (PSB-603)), and their affinities for the different adenosine receptor subtypes [61,62]. (**B**) Twenty-four hours after transfection, the cells were pre-treated for 30 min with the selective A_2B_AR antagonist PSB-603 1 µM or 1% DMSO at 37 °C in OptiMEM medium. Then the cells were stimulated for 15 min with the non-selective adenosine receptor agonist NECA (10 µM), the selective A_2B_AR partial agonist BAY-60-6583 (10 µM), and the endogenous adenosine receptor agonist adenosine (100 µM). The NanoGlo live-cell substrate was added, and the association was immediately monitored at 25 °C over a time period of 42 min, with a luminescence reading taken every 30 s. (**C**) After a time period of ~ 700 s, the maxima of complex formation were reached, and the relative luminescence units (RLU) were plotted. Significant differences were observed between the untreated LgBiT-GGTase-I-β/FTase α/SmBiT-Rap1B complex and the NECA and adenosine treated Lg-BiT-GGTase-I-β/FTase α/SmBiT-Rap1B complex (* *p* < 0.05, ** *p* < 0.01). No significant differences were observed between the untreated LgBiT-GGTase-I-β/FTase α/SmBiT-Rap1B complex and the PSB-603 and BAY60-6593 treated Lg-BiT-GGTase-I-β/FTase α/SmBiT-Rap1B complex (ns, not significant). An unpaired two-tailed Welch’s *t*-test was used to compare the 10 µM NECA treated cells versus 10 µM NECA + 1 µM PSB-603 treated cells (ns, not significant).

To investigate this further, we examined the effect of A_2B_AR-selective and non-selective agonists and antagonists on the interaction of GGTase-I and Rap1B in HEK293 cells endogenously expressing A_2B_ARs and A_2A_ARs and recombinant A_2B_ARs (Figure 4A, Supplementary Figures S5 and S6) [41,43,44,49]. The non-selective adenosine receptor agonists adenosine 100 µM, 5′-*N*-ethylcarboxamidoadenosine (NECA) 10 µM, and the A_2B_AR-selective partial agonist [2-({6-amino-3,5-dicyano-4-[4-(cyclopropylmethoxy)phenyl]pyridin-2-yl}sulfanyl)acetamide] (BAY60-6583) 10 µM were selected in concentrations that achieve maximal intrinsic efficacies in cAMP accumulation assays in HEK293 cells stably expressing A_2B_ARs [43]. The concentration of the selective A_2B_AR antagonist 8-(4-(4-(4-chlorophenyl)piperazine-1-sulfonyl)phenyl)-1-propylxanthine (PSB-603) 1 µM was chosen regarding its maximal A_2B_AR blocking effect (*K*_i_ = 0.553 nM) but still preserving selectivity versus A_2A_AR (*K*_i_ > 10,000 nM) (Figure 4A) [60]. The AR ligand treated complex LgBiT-GGTase-I-β-subunit/FTase α-subunit/SmBiT-Rap1B WT showed similar kinetic properties as the untreated (2% DMSO) LgBiT-GGTase-I-β-subunit/FTase α-subunit/SmBiT-Rap1B WT complex (Figure 4B). Incubation with the non-selective AR receptor agonists NECA (10 µM) and adenosine (100 µM) significantly stimulated the interaction of GGTase-I with Rap1B (* *p* < 0.05, ** *p* < 0.01) (Figure 4C). Moreover, also forskolin (10 µM) significantly increased the interaction of GGTase-I with Rap1B (* *p* < 0.05, Supplementary Figure S7), and its effect was comparable to that of NECA and adenosine (Supplementary Figure S7). The selective A_2B_AR partial agonist BAY60-6583 (10 µM) and the selective A_2B_AR antagonist 8-(4-(4-(4-chlorophenyl)piperazine-1-sulfonyl)phenyl)-1-propylxanthine (PSB-603) (1 µM) alone had no influence on GGTase-I/Rap1B complex formation (ns, not significant) (Figure 4C). Pre-incubation with the A_2B_AR selective antagonist PSB-603 1 µM inhibited the response of 10 µM NECA, but the difference was not significant (Figure 4C). 

### 2.5. Effect of A_2A_AR Agonists and Antagonists on the Interaction of GGTase-I and Rap1B

**Figure 5:**
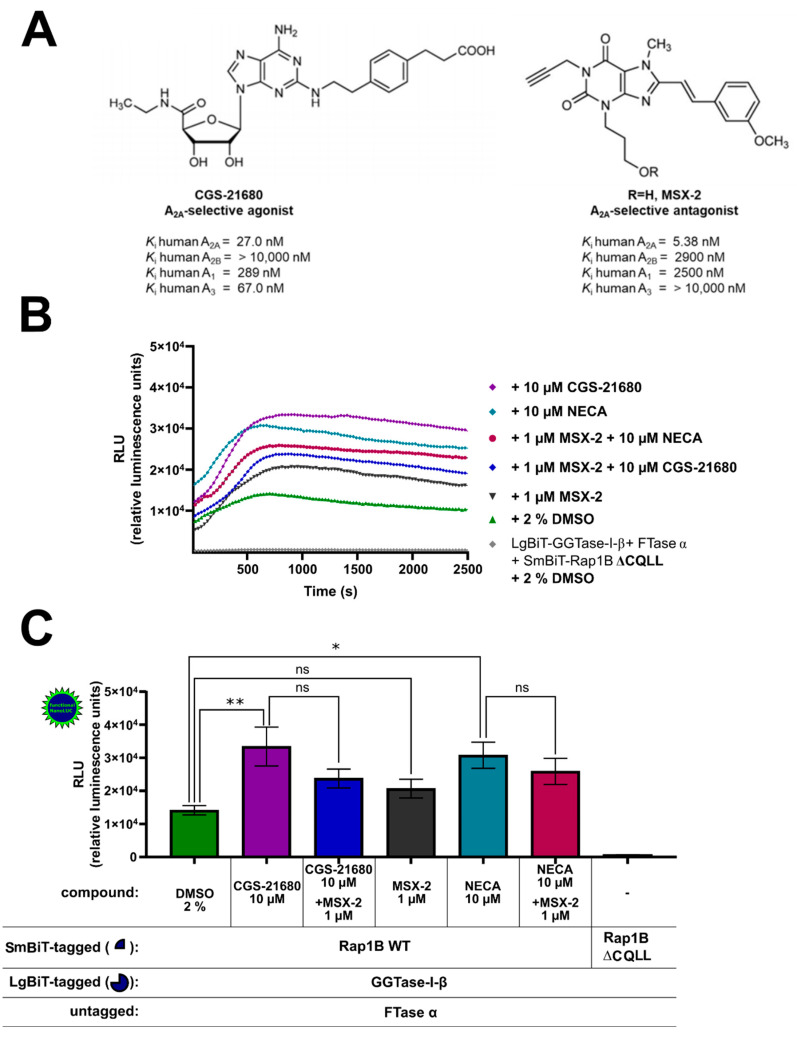
Effect of A_2A_AR agonists and antagonists on the interaction of GGTase-I and Rap1B. HEK293 cells stably expressing the A_2B_AR were transiently transfected as described above. (**A**) Selected A_2A_AR-selective agonist 2-[*p*-(2-carboxyethyl)phenylethyl-amino]-5′-*N*-ethylcarboxamido adenosine (CGS-21680) and A_2A_AR-selective antagonist (3-(3-hydroxypropyl)-7-methyl-8-(*m*-methoxystyryl)-1-propargylxanthine) (MSX-2) and their affinities at the different adenosine receptor subtypes [61,62]. (**B**) Twenty-four hours after transfection, the cells were pre-treated for 30 min with the selective A_2A_AR antagonist MSX-2 (1 µM) or 1% DMSO at 37 °C in OptiMEM medium. Then the cells were stimulated for 15 min with the selective A_2A_AR agonist CGS-21680 (10 µM) and the non-selective adenosine receptor agonist NECA (10 µM). The NanoGlo live-cell substrate was added, and the association was immediately monitored at 25 °C over a time period of 42 min, with a luminescence reading taken every 30 s. (**C**) After a time period of ~ 700 s, the maxima of complex formation were reached, and the relative luminescence units (RLU) were plotted. Significant differences were observed between the untreated LgBiT-GGTase-I-β/FTase α/SmBiT-Rap1B complex and the 10 µM NECA and 10 µM CGS-21680 treated Lg-BiT-GGTase-I-β/FTase α/SmBiT-Rap1B complexes (* *p* < 0.05, ** *p* < 0.01). No significant differences were observed between the untreated LgBiT-GGTase-I-β/FTase α/SmBiT-Rap1B complex and the 1 µM MSX-2 treated Lg-BiT-GGTase-I-β/FTase α/SmBiT-Rap1B complexes (ns, not significant). An unpaired two-tailed Welch’s *t*-test was used to compare the 10 µM CGS-21680 treated cells versus 10 µM CGS-21680 + 1 µM MSX-2 and 10 µM NECA treated cells versus 10 µM NECA + 1 µM MSX-2 (ns, not significant).

HEK293 cells express both endogenous Gα_S_-coupled A_2A_AR and A_2B_AR, and their activation increases intracellular cAMP levels [49], which results in PKA activation and might influence the Rap1B phosphorylation and prenylation. To investigate the effect of A_2A_AR signaling on the interaction of GGTase-I and Rap1B, the A_2A_AR specific agonist 2-[*p*-(2-carboxyethyl)phenylethyl-amino]-5′-*N*-ethylcarboxamido adenosine (CGS-21680) 10 µM and the A_2A_AR specific antagonist (3-(3-hydroxypropyl)-7-methyl-8-(*m*-methoxystyryl)-1-propargylxanthine) (MSX-2) 1 µM were utilized. CGS-21680 exhibits high affinity at human A_2A_AR (*K*_i_ = 27.0 nM) and high selectivity versus A_2B_AR (*K*_i_ > 10,000 nM) [62]. The A_2A_AR antagonist MSX-2 exhibits a *K*_i_-value of 5.38 nM at human A_2A_AR and a high selectivity versus A_2B_AR (*K*_i_ = 2900 nM) (Figure 5A) [62]. The AR ligand-treated complex formation LgBiT-GGTase-I-β-subunit/FTase α-subunit/SmBiT-Rap1B WT showed similar kinetic properties as the untreated (2% DMSO) LgBiT-GGTase-I-β-subunit/FTase-α-subunit/SmBiT-Rap1B WT complex formation (Figure 5B). Incubation with the non-selective AR receptor agonist NECA (10 µM) or with the A_2A_AR agonist CGS-21680 (10 µM) significantly stimulated the interaction of GGTase-I with Rap1B (* *p* < 0.05 and ** *p* < 0.01, respectively) (Figure 5C). The selective A_2A_AR antagonist MSX-2 (1 µM) alone had no significant influence on GGTase-I/Rap1B interaction (Figure 5C). Pre-incubation with the A_2A_AR selective antagonist MSX-2 (1 µM) inhibited the response of 10 µM CGS-21680 and slightly the response of 10 µM NECA, but the difference was not significant (Figure 5C). 

## 3. Discussion

GGTase-I represents an important drug target due to its involvement in different kinds of pathological processes [2,14,15]. In particular, GGTase-I inhibitors have attracted interest regarding their application in different types of cancer [18,22,55,63,64]. In this study, we developed a NanoBiT assay to measure the interaction of Rap1B and human GGTase-I, which is expected to become a pharmacological tool for the evaluation of novel GGTI inhibitors. The assay depends upon the expression of proteins tagged with the LgBiT and SmBiT reporters. Despite observing a robust luminescence signal for LgBiT-GGTase-I-β/SmBiT-FTase α and LgBiT-GGTase-I/FTase α/SmBiT-Rap1B, we were not able to quantify the expression of all tagged proteins by immunoblotting. However, as shown in the Western blots, all DNA constructs were clearly expressed in HEK293 cells. Especially, the negative control LgBiT-FTase α/SmBiT-FTase α was clearly expressed, although it elicited only a moderate luminescence signal, suggesting that the assay is specific and not a result of the overexpressed NanoBiT tags associating with each other. Secondly, the LgBiT-GGTase-I-β co-transfected with HaloTag-SmBiT^®^, which was not expected to interact, showed only a low formation of functional NanoLuc. Moreover, the luminescence signal of LgBiT-GGTase-I-β/FTase α/SmBiT-Rap1B was reduced by the cell-permeable, competitive, and selective peptidomimetic CAAX motif inhibitor GGTI-298. GGTI-298 was proven to inhibit the processing of the structural homolog geranylgeranylated Rap1A with an IC_50_-value of 3 µM [52,53]. Toxic effects on HEK293 cells or inhibition of the functional NanoLuc can also be excluded, since GGTI-298 had no effect on the formation of A_2A_AR-homodimers. Furthermore, the LgBiT and SmBiT subunits only weakly associate with each other, and their intrinsic affinity (*K*_D_ = 190 µM) is outside of the ranges typical for protein interactions [23]. Thus, we conclude that the assay offers a new pharmacological tool to perform close to real-time measurements and to monitor reversible protein–protein interactions. In this format, it depends on transient transfection and could also be used for different cancer cell lines. However, it would be useful to establish stable cell lines with constant expression ratios for better comparability of IC_50_-values for tested GGTase-I inhibitors.

Despite the number of solved crystal structures, many aspects of enzyme substrate specificity and allosteric modulation of enzyme activity still remain unclear. So far, there is no crystal structure of the human GGTase-I in complex with its substrate Rap1B available, and understanding the key features of substrate specificity will contribute to optimization of anti-cancer drugs. In order to obtain further molecular insights into the interaction between human GGTase-I and its substrate Rap1B, we substituted the cysteine residue (C) in the CQLL motif for serine (S) or glycine (G). The prenylation-deficient mutants Rap1B GQLL and SQLL were obtained. Moreover, a complete CAAX motif deletion mutant, ΔCQLL, was generated. HEK293 cells stably expressing the A_2B_AR were transiently co-transfected either with LgBiT-GGTase-I-β-subunit/FTase α-subunit/SmBiT-Rap1B WT or with LgBiT-GGTase-I-β-subunit/FTase α-subunit in combination with the SmBiT-Rap1B prenylation-deficient mutants C181G, C181S, and ΔCQLL. Interestingly, both Rap1B mutants C181G and C181S were still able to fully interact with GGTase-I, whereas the mutant missing the CAAX motif showed a decreased interaction with the enzyme.

In general, the prenylation reaction begins when GGTase-I binds its isoprenoid substrate geranylgeranylpyrophosphate (GGPP) and forms a binary enzyme diphosphate complex [56]. GGPP is a key intermediate in the isoprenoid biosynthesis pathway, and its concentration, e.g., in different human pancreatic cancer cell lines, varies from 1.96 nmol/10^6^ cells to 9.96 nmol/10^6^ cells [65]. After isoprenoid binding, the CAAX protein binds and its cysteine residue coordinates to a Zn^2+^ ion (as a thiolate) in the active site of the enzyme, which is necessary for its catalytic activity [3,7,56,66]. Additionally, the Zn^2+^ ion is coordinated by three conserved residues, D269β, C271β, and H321β, in GGTase-I [66]. Further, the cysteine thiolate forms a covalent thioether linkage to the isoprenoid at the C-1 position. Upon binding of new GGPP, the prenylated protein product is released from the enzyme, which is the rate-limiting step [56]. Regarding the enzyme’s mechanism, our designed prenylation-deficient Rap1B mutants C181G and C181S were not able to form the bonded Zn^2+^-to-cysteine-thiolate interaction with GGTase-I, but we were still able to measure an interaction. It appears that the remaining three residues in the CAAX motif QLL (glutamine, leucine, leucine) are sufficient to stabilize the interaction with the enzyme. Modeling studies revealed that polar or charged a_1_ residues in the Ca_1_a_2_X motif could form direct or water-mediated hydrogen bonds with rat GGTase-I and thereby enhance binding affinity [7]. The polar glutamine (Q) in the a_1_ position of Rap1B might react in a similar way with human GGTase-I. Moreover, in rat GGTase-I the a_2_ (isoleucine) side chain makes extensive hydrophobic contacts with Phe53β and Leu320β, as well as the fourth isoprene unit [3]. The same could be the case for the a_2_ leucine residue in Rap1B. The X residue in the Ca_1_a_2_X motif is the primary determinant specifying whether the peptide is a substrate for FTase or for GGTase-I. GGTase-I harbors only one binding pocket for X residues, which is shaped to accommodate hydrophobic residues and discriminates against polar or charged residues [7]. It is obvious that the X leucine residue of Rap1B fits inside the hydrophobic binding pocket and contributes to the binding affinity to GGTase-I. In Western blots, our generated Rap1B ΔCQLL mutant exhibited a ~ 5-fold lower expression level compared to the Rap1B WT protein and the mutants C181G and C181S. Additionally, the interaction with GGTase-I was more than 20-fold reduced compared to Rap WT interaction with GGTase-I, but the generated luminescence of ΔCQLL/GGTase-I was still higher (800–1000 RLU, Figure 2C and Figure 3B) when compared to the clearly expressed negative control LgBiT-FTase/SmBiT-FTase alpha (200–300 RLU, Figure 1B,D).

Thus, the diminished interaction of ΔCQLL mutant with GGTase-I might not only be a result of lacking expression but could also be a result of a decreased binding affinity to the enzyme. This point would be supported by the presented observation that all CAAX motif residues are involved in the binding affinity to the enzyme. On the other hand, we could also not exclude that other amino acid residues of Rap1B are involved in the binding to GGTase-I.

In order to obtain further insights into the role of A_2A_AR and A_2B_AR signaling on the GGTase-I and Rap1B interaction, we investigated whether selected specific and non-specific A_2A_AR and A_2B_AR agonists and antagonists exhibit a pharmacological effect. Our results demonstrated that the non-selective agonists adenosine (100 µM), the metabolically stable adenosine analog derivative NECA (10 µM), and the selective A_2A_AR agonist CGS-21680 (10 µM) showed a significant increase on human GGTase-I and Rap1B interaction. Additionally, forskolin (10 µM), which directly activates adenylate cyclase, showed similar effects when compared to adenosine (100 µM) and NECA (10 µM). In cAMP experiments at HEK293-A_2B_ cells, NECA (10 µM) showed a higher cAMP effect when compared to adenosine (100 µM) and forskolin (10 µM) (Supplementary Figure S5), but cAMP assays might not correlate with a much further downstream signal like GGTase-I/Rap1B interaction. In contrast, the selective A_2B_AR partial agonist BAY60-6583 (10 µM) was not able to increase the interaction in the utilized HEK-A_2B_ cell line. The selective A_2B_AR antagonist PSB-603 (1 µM) was able to reduce the interaction stimulated by 10 µM of NECA, but the difference was not significant. It is likely that the residual response elicited by 10 µM NECA is due to an endogenous A_2A_AR population in HEK293 cells [41,49] since the specific A_2A_AR agonist CGS-21680 (10 µM) also produced an increase in luminescence. The selective A_2B_AR antagonist PSB-603 (1 µM) or the selective A_2A_AR antagonist MSX-2 (1 µM) alone had no significant effect on the interaction, suggesting a relatively low endogenous adenosine concentration in HEK293 cells. The A_2A_AR specific antagonist MSX-2 (1 µM) showed only a moderate but not significant decrease in the interaction elicited by the non-selective agonist NECA (10 µM) or the specific adenosine A_2A_AR agonist CGS-21680 (10 µM), suggesting a relatively low A_2A_AR population in HEK293 cells, which was previously shown by qRT-PCR [41].

Our results and previous studies demonstrate that A_2A_AR forms homodimers or higher order oligomers, which may cause allosteric modulation and cooperativity [53,67]. For example, binding of the first ligand induces a conformational change in the A_2A_-D_2_ (dopamine D_2_ receptor) heterotetramer and will then reduce the affinity of the second ligand [68]. Another explanation would involve the presence of different A_2A_AR receptor conformations [69], which probably bind agonists and antagonists with different affinity. Based on these results, we speculate that regarding the observed pharmacology, different portions of A_2B_AR/A_2A_AR monomer/homodimer/oligomer populations might be involved in the increased interaction of GGTase-I with Rap1B. Alternatively, the relatively high concentration of 10 µM CGS-21680 might additionally activate A_2B_ARs in HEK293 cells, which could explain that the specific A_2A_AR specific antagonist MSX-2 (1 µM) could not fully reduce the luminescence signal elicited by CGS-21680.

Regarding the prenylation and the function of Rap1B, we can assume that an increase in the interaction of human GGTase-I with Rap1B might at least modulate the prenylation and localization of Rap1B. In earlier studies, Ntantie et al. demonstrated that A_2B_AR activation with NECA increased total Rap1B protein but delayed its prenylation and localization in the plasma membrane [45,46]. Additionally, it was shown that NECA induced phosphorylation of S179 and S180 in the PBR region of Rap1B and inhibited the binding to the chaperone protein Smg-GDS-607, which was suggested to bind non-prenylated small GTPases and to assist in their prenylation [46]. On the other hand, Garcia-Torres et al. reported a dual role for Smg-GDS-607 activating and inhibiting farnesylation of small GTPases [70]. For example, they discovered that SmgGDS-607 increased the rate of farnesylation of H-Ras by enhancing product release from FTase [70]. One explanation for the increased GGTaseI/Rap1B formation induced by A_2A_AR/A_2B_AR signaling in the present study might be that the decreased binding of Smg-GDS-607 to Rap1B led to delayed product release of prenylated Rap1B. This could result in increasing complex formation and thereby a lower accumulation of prenylated Rap1B at the cell membrane, which is consistent with the studies of Ntantie et al. [45].

Finally, we can conclude that both A_2A_AR and A_2B_AR activation contributes to an increased interaction of GGTase-I with Rap1B. A greater understanding of the molecular mechanism of cancer development will lead to the identification of novel therapeutics and better treatments. Regarding the model that increased Rap1B geranylgeranylation and thereby increased cell adhesion decreases metastasis, A_2A_AR and A_2B_AR antagonists are promising candidates for therapeutic intervention in different types of cancer that overexpress Rap1B.

## 4. Materials and Methods

### 4.1. Material

Dulbecco’s Modified Eagle Medium (DMEM), penicillin-streptomycin solution, and all cell culture supplements were purchased from Pan-Biotec (Aidenbach, Germany). Fetal calf serum (FCS) and geneticin (G418) were obtained from Sigma (St. Louis, MI, USA) and Roth (Karlsruhe, Germany). All chemicals were purchased from Roth (Karlsruhe, Germany), Sigma (St. Louis, MI, USA), or Selleckchem (Houston, TX, USA) unless otherwise noted. The enzymes for molecular biology were purchased from Thermo Fisher Scientific (Waltham, MA, USA) or Promega (Madison, WI, USA). All primers were obtained from Eurofins Scientific (Hamburg, Germany). The vectors pFN217k LgBiT CMV-Hyg Flexi, pFN218k SmBiT CMV-Blast Flexi, pFC219k LgBiT CMV-Hyg Flexi, pFC220k SmBiT CMV-Blast Flexi, HaloTag^®^-SmBiT and the NanoGlo substrate were purchased from Promega (Madison, MA, USA). Q5 DNA-polymerase was obtained from New England Biolabs (Frankfurt, Germany). Lipofectamine 3000 for transfection was purchased from Thermo Fisher Scientific (Waltham, MA, USA). All solutions for the Western blots were obtained from LI-Cor Biosciences (Lincoln, USA). All antibodies were obtained from Abcam (Cambridge, UK) or LI-Cor Biosciences (Lincoln, NE, USA) unless otherwise noted. The specific anti-LgBiT/NanoLuc antibody was kindly provided by Promega (Madison, USA). The cDNA encoding for the human Rap1B (NM_001010942.3) was kindly received from Prof. Jun Qin, Department of Molecular Medicine, School of Medicine, Cleveland Clinic. The plasmid pcDNA 3.1 (+)-FTase β (NM_002028.4) and the cDNAs of GGTase-I-β (NM_005023.4) and FTase α (NM_002027.3) in pETDuet1 plasmid were already available [5,71]. The adenosine A_2A_- and A_2B_-receptor agonists and antagonists, the vector pcDNA3.1(−), the vector pcDNA3.1(−)-HA-A_2A_AR (NM_000675.6), and the established HEK293 cell line stably expressing the human A_2B_AR (NM_000676.4) [43] were a gift from the University of Bonn from Prof. Dr. Christa E. Müller’s research group. [^3^H]cAMP (3′,5′-cyclic adenosine monophosphate; specific activity 34 Ci/mmol) for cAMP assays was utilized at Prof. Dr. Christa E. Müller’s research group.

### 4.2. Expression Vectors and Molecular Cloning

The human Rap1B WT and mutants C181G, C181S, ΔCQLL, the human GGTase-I-β-subunit, and the human FTase α-subunit coding regions were subcloned into the *N*-terminal Flexi vectors pFN217k LgBiT CMV-Hyg Flexi, pFN218k SmBiT CMV-Blast Flexi containing *SgfI* and *PmeI* sites. The following primers were used:

- f-Rap1B WT-Sgf1: 5′-ggacGCGATCGCCCGTGAGTATAAGCTAGTCGTTC
- r-Rap1B WT-PmeI: 5′-gtccGTTTAAACTTAAAGCAGCTGACATGATGAC
- r-Rap1B-(C181G)-PmeI: 5′-gtccGTTTAAACTTAAAGCAGCTGACCTGATGAC
- r-Rap1B-(C181S)-PmeI: 5′-gtccGTTTAAACTTAAAGCAGCTGACTTGATGAC
- r-Rap1B-(ΔCQLL)-PmeI: 5′-gtccGTTTAAACTTATGATGACTTTTTGCGAGCC
- f-GGTase-I-β-Sgf1: 5′-ggacGCGATCGCCGCGGCCACTGAGGATGAGAG
- r-GGTase-I-β-PmeI: 5′-gtccGTTTAAACTCATGTGGAGATATGTACATTCTC
- f-FTase α-Sgf1: 5′-ggacGCGATCGCCGCGGCCACCGAGGG
- r-FTase α-PmeI: 5′-gtccGTTTAAACTTATTGCTGTACATTTGTTGGTG.

The following PCR program was applied: 30 s at 98 °C and 30 cycles consisting of 10 s at 98 °C, 30 s at 60–63 °C, and 2 min at 72 °C, followed by a final elongation step of 2 min at 72 °C. The PCR products were purified and digested with *SgfI* and *PmeI.* The *N*-terminal Flexi vectors pFN217k LgBiT CMV-Hyg Flexi, pFN218k SmBiT CMV-Blast Flexi were cut with *SgfI* and *PmeI* and, after ligation, *N*-terminal fusion proteins were created, allowing translational readthrough of the *SgfI* side, which encodes the peptide sequence Ala-Ile-Ala. The correct assembly of the genes was verified by sequencing (Seqlab, Göttingen, Germany) using the following primers:

- pFN217k LgBiT→forward: 5′ GGAACGGCAACAAAATTATCGAC
- pFN217k LgBiT→reverse: 5′ CATGCCTGCAGGTCGACTCTAG
- pFN218k SmBiT→forward: 5′ CAGTTCAATTACAGCTCTTAAGGCTAGAG
- pFN218k SmBiT→reverse: 5′ CAGCTTGCATGCCTGCAG

The cDNA of the A_2A_AR was subcloned with *SgfI* and *PmeI* without a stop codon into the *C*-terminal Flexi vectors pFC219k LgBiT and pFC220k SmBiT, which were cut with *SgfI* and *EcoICRI*. The following primers were designed:

- f-hA_2A_-*SgfI*: 5′-ggacGCGATCGCCATGCCCATCATGGGCTCCTC
- r-hA_2A_-*PmeI*: 5′-gtccGTTTAAACGGACACTCCTGCTCCATCCTG

The PCR was conducted as described above. *C*-terminal fusion proteins were created by fusing the blunt-cut *PmeI* end of the protein coding region with the blunt end generated by *EcoICRI*. The correct assembly of the genes was verified by sequencing (Seqlab, Göttingen, Germany) using the following primers:

- pFC219K LgBiT→forward: 5′ CTGGTTTAGTGAACCGTCAGATC
- pFC219K LgBiT→reverse: 5′ GCAATACGTCGACGTTATCAGCTG
- pFC220K SmBiT→forward: 5′ CAGTTCAATTACAGCTCTTAAGGCTAGAG
- pFC220K SmBiT→reverse: 5′ CAGCTTGCATGCCTGCAG

The following fusion proteins were received and tested in different plasmid amounts in the NanoBiT assay: SmBiT-Rap1B WT, SmBiT-Rap1B C181G, SmBiT-Rap1B C181S, SmBiT-Rap1B ΔCQLL, LgBiT-GGTase-I-β, LgBiT-FTase α, SmBiT-FTase α, A_2A_-LgBiT, A_2A_-SmBiT. For the expression of the untagged human FTase α-subunit, the cDNA was subsequently subcloned into the vector pcDNA3.1 (−). The coding region of human FTase *α-*subunit was amplified via PCR using Phusion-High-Fidelity-DNA-Polymerase (NEB, Ipswitch, USA). The following primers were designed:

- f-: TTTGAATTCATGGCGGCCACCGAG
- r-: AAAGGATCCTTATTGCTGTACATTTGTTGG.

The PCR program was conducted as follows: 3 min at 98 °C and 30 cycles consisting of 30 s at 98 °C, 45 s at 58 °C, and 2 min at 72 °C, followed by a final elongation step of 10 min at 72 °C. The PCR product was purified and digested with *EcoRI* and *BamHI* and finally cloned into the vector pcDNA3.1 (−). The plasmids were transformed into competent *Escherichia coli DH5α* and single clones were isolated and sequenced (Seqlab, Göttingen, Germany).

### 4.3. Cell Culture

HEK293 stably expressing the human A_2B_AR were maintained in DMEM supplemented with 4.5 g/L *D*-glucose, 10% heat inactivated FCS, 100 U/mL of penicillin, 100 μg/mL streptomycin, and 800 µg/mL G418. The cells were cultured in an incubator with an atmosphere containing 5% CO_2_ at 37 °C and were directly transfected in white 96-well plate format.

### 4.4. Transient Transfection of HEK293 Cells Stably Expressing the Human A_2B_AR

Fifty thousand cells/well were seeded, resulting in ~ 80% confluency at the time of transfection. Two hours before the transfection, the cell culture medium was changed to 100 µL of OptiMEM medium. For duplicates, 9 µL of OptiMEM medium without supplements was mixed to 1 µL of Lipofectamine 3000 reagent. Fifty to two hundred nanograms of plasmid constructs were mixed with OptiMEM medium and 1 µL of P3000 reagent to reach a final volume of 10 µL. The lipofectamine suspension was added and mixed to the DNA solution, incubated for 15 min at rt, and 10 µL of the transfection mix was finally added to the wells. After 20 h the cell culture medium was changed to 100 µL of fresh OptiMEM medium and further incubated for 4 h at 37 °C before performing the experiments.

### 4.5. NanoBiT Assays

For a first orientation screen, cells were transfected with 50 ng/well of each LgBiT and SmBiT plasmid alone or in combination with the investigated partner protein. The combinations LgBiT-FTase α/SmBiT-FTase α and LgBiT-GGTase-I-β/HaloTag-SmBiT^®^ were used as negative controls and the combination LgBiT-GGTase-I-β/SmBiT-FTase α as a positive control. The NanoGlo live-cell reagent containing the cell-permeable furimazine substrate was dissolved 1:50 in NanoGlo dilution buffer or in OptiMEM medium, and 25 µL of the solution was added to each well. The luminescence was immediately monitored at 25 °C over a time period of up to 60 min with a luminescence reading taken every 30 s using a Tecan microplate reader (Tecan group Ltd., Männedorf, Switzerland).

### 4.6. Treatment with the Cell-Permeable and Selective GGTase-I Inhibitor GGTI-298

The cells were seeded as described above. The transfection pairs used were LgBiT-GGTase-I-β/FTase α/SmBiT-Rap1B WT, A_2A_-LgBiT/A_2A_-SmBiT, and LgBiT-GGTase-I-β/FTase α/SmBiT-Rap1B ΔCQLL as a negative control, with transfection preparation as described above. Twenty-four hours after the transfection, the cells were pre-treated for 30 min with either the selective GGTase-I inhibitor GGTI-298 (final concentration 10 µM, 1 µM, 0.1 µM, 0.01 µM) or vehicle (DMSO 1% final concentration) at 37 °C in OptiMEM medium. Then 25 µL of the NanoGlo live-cell substrate was added and the association was immediately monitored at 25 °C over a time period of 30–50 min, with a luminescence reading taken every 30 s.

### 4.7. Treatment with Adenosine A_2A_- and A_2B_-Receptor Agonists and Antagonists

Cells were seeded and transfected as described above. After 24 h, the cells were pre-treated for 30 min with the selective A_2A_AR antagonist MSX-2 (1 µM), the selective A_2B_AR antagonist PSB-603 (1 µM), or vehicle (1% DMSO) at 37 °C in OptiMEM medium. Then the cells were stimulated for 15 min with the non-selective adenosine receptor agonist NECA (10 µM), the selective adenosine A_2A_ receptor agonist CGS-21680 (10 µM), the selective adenosine A_2B_ receptor partial agonist BAY-60-6583 (10 µM), or with the endogenous adenosine receptor agonist adenosine (100 µM). The final DMSO concentration did not exceed 2%. Then 25 µL of the NanoGlo live-cell substrate was added and the association was immediately monitored at 25 °C over a time period of 42 min, with a luminescence reading taken every 30 s.

### 4.8. Transient Transfection of HEK-A_2B_ Cells for Western Blot Analysis

In 6-well plates, 3 × 10^5^ cells were seeded, resulting in ~ 90% confluency at the time of transfection. Two hours before the transfection, the cell culture medium was changed to 2 mL of OptiMEM medium. One hundred and fifteen microliters of OptiMEM medium without supplements was mixed to 10 µL of Lipofectamine 3000 reagent. Three micrograms of pcDNA3.1 (−)—FTase α/2 µg pcDNA3.1 (+) FTase β, 3 µg of LgBiT-GGTase-I-β/2 µg FTase α, 3 µg LgBiT-FTase α/2 µg FTase β, 3 µg SmBiT-FTase α/2 µg FTase β, 4 µg of SmBiT-Rap1B WT, SmBiT-Rap1B C181G, SmBiT-Rap1B C181S, and SmBiT-Rap1B ΔCQLL were mixed with OptiMEM medium and 5 µL of P3000 reagent to reach a final volume of 125 µL. The lipofectamine suspension was added and mixed to the DNA solution, incubated for 10 min at rt, and finally added to the cells. After 16 h, the cell culture medium was changed to 2 mL of growth medium and further incubated for 8 h at 37 °C before harvesting the cells for Western blot analysis.

### 4.9. Preparation of Cytosolic Extract and Whole Cell Lysate of Transfected HEK-A_2B_ Cells

The transfected cells were harvested and disrupted by repeated aspiration at 4 °C through a 500 µL and afterwards a 10 µL pipette tip in 250 µL of RIPA-buffer containing 150 mM NaCl, 1% NP-40, 0.1% SDS, 0.5% sodium deoxycholate, 25 mM TRIS, pH 7.4, and protease inhibitor. The cytosolic extract was obtained by centrifugation at 20,000× *g* for 2 h at 4 °C. For the A_2B_AR Western blots, the whole cell lysate was used, or it was purified by centrifugation at 1000× *g* for 3 min. The protein concentration of the supernatant fraction was determined, and different protein amounts (20–80 µg) were used for the Western blots.

### 4.10. Bradford Protein Determination

The protein concentration of the cytosolic extract was determined by the Bradford assay. A bovine serum albumin (BSA) standard in the range of 1 to 15 µg/mL was diluted in H_2_O to a final volume of 600 µL. Additionally, 1 µL of the samples were diluted in 599 µL of H_2_O. Then, 400 µL of Bradford reagent (Bio-Rad, Berkeley, USA) was added to the different standard concentrations, and the diluted samples and the absorbance were measured at 595 nm in a standard photometer.

### 4.11. SDS-Page and Western Blotting

Cytosolic fractions of transfected cells were analyzed by sodium dodecyl sulfate-polyacrylamide gel electrophoresis (SDS-PAGE) and Western blots with specific first monoclonal FTase α antibody (1:1500) and infrared second antibody (IRDye^®^800CW goat anti-rabbit IgG, 1:10,000) to detect untagged FTase *α* (~44.4 kDa), LgBiT-FTase α (~63.9 kDa) and SmBiT-FTase α (~47.7 kDa). A specific first polyclonal Rap1B antibody (1:1000, anti-rabbit IgG) (Proteintech, Manchester, UK) and infrared second antibody (1:10,000, IRDye^®^800CW, goat anti-rabbit IgG) were used to detect SmBiT-Rap1B (~24 kDa). To detect the LgBiT, a specific first anti-rabbit NanoLuc antibody (1:5000) was provided by Promega (Madison, USA), and infrared second antibody (1:10,000, IRDye^®^800CW, goat anti-rabbit IgG) was used to detect the fusion protein LgBiT-GGTase-I-β. A specific first monoclonal GAPDH antibody (1:2000, anti-mouse igG1, kappa, Thermo Fisher scientific) and infrared second antibody (1:10,000, IRDye^®^ 680RD donkey anti-mouse IgG) were used to detect GAPDH (~37 kDa) as a loading control.

Whole cell lysate of HEK-A_2B_ cells was analyzed by sodium dodecyl sulfate-polyacrylamide gel electrophoresis (SDS-PAGE) and Western blots with specific first polyclonal A_2B_AR antibody (1:1000) and infrared second antibody (IRDye^®^800CW goat anti-rabbit IgG, 1:10,000) to detect A_2B_AR (~37 kDa).

After semidry blotting for 30 min and 25 V, the nitrocellulose membrane was blocked in blocking solution for 90 min at rt. Then, the membrane was incubated with the specific first antibodies, which were diluted in LI-Cor antibody solution, 0.1% Tween 20, for 60 min at rt. Afterward, the membrane was washed 4 times for 10 min with Tris-buffered saline (TBS), 0.1% Tween 20, and then incubated with the second infrared antibodies for 60 min at rt and shaking in the dark. Finally, the membrane was washed 3 times for 10 min with TBS, 0.1% Tween 20, and 5 min with TBS, dried in the dark, and visualized on an Odyssey imager (LI-Cor, Lincoln, USA). ImageJ was used for quantification of Western blot signals. At least 3 independent experiments were performed in single values for quantification, and data were presented as mean ± SEM.

### 4.12. cAMP Accumulation Experiments

The assays were conducted at the University of Bonn, Pharmaceutical Institute, Pharmaceutical and Medicinal Chemistry. cAMP accumulation experiments at HEK-A_2B_ cells were performed according to De Filippo et al. [72] with the following modifications. HEK-A_2B_ cells were detached from a confluent 175 cm^2^ flask and centrifuged at 200× *g* at 4 °C for 5 min. Then the supernatant was removed, and the cell pellet was resuspended in Hanks’ balanced salt solution (HBSS) buffer, pH 7.4 with 1 U/mL adenosine deaminase (ADA). When adenosine was used, ADA was omitted. Two hundred microliters of the cell solution containing ~ 200,000 cells were transferred into 24-well plates. After an incubation time of 3 h at 37 °C, 5% CO2, 25 µL of the phosphodiesterase inhibitor Ro20-1724 (40 µM) dissolved in 100% HBSS buffer was added to each well. After an incubation time of 10 min, 12.5 µL of the antagonists were added and incubated for 30 min at 37 °C. Then, 12.5 µL of the agonists were added and incubated for 15 min at 37 °C. The final DMSO concentration did not exceed 1.4%. cAMP accumulation was stopped by the addition of 250 µL of hot lysis buffer (90 °C, 8 mM EDTA, 0.02% Triton X-100, pH 7.3). The 24-well plates were put on ice, and each well was subsequently homogenized. Fifty microliters of each cell lysate were transferred in 2.5 mL tubes. Radioligand competition binding experiments with [³H]cAMP to determine the cAMP amount in the lysates were conducted according to De Filippo et al. [72].

### 4.13. Statistical Analysis

For all statistically analyzed studies, at least 3 independent experiments were performed, each in duplicates unless otherwise stated. Data were expressed as mean ± SEM and differences with *p* < 0.05 were considered significant. One-way ANOVA followed by Dunnett’s post hoc test was used to evaluate the differences among three or more groups. In order to determine the differences between two groups, an unpaired parametric Welch’s *t*-test was employed. GraphPad prism 8.0 was used for data analysis and statistics. Inkscape was employed for graphical representations.

## 5. Conclusions

The new NanoBiT assay might become a pharmacological tool for the evaluation of novel GGTase-I inhibitors. Moreover, both A_2A_AR and A_2B_AR activation contribute to an increased interaction of GGTase-I with Rap1B. In both cases, it has to be considered that prenylation of Rap1B is not always required for its oncogenic activity, and additionally, Rap1B prenylation and function appear to be cell-type dependent.

## Data Availability

All relevant data are within the manuscript and its Supporting Information files.

## Supplementary Materials

The following are available online at https://www.mdpi.com/1422-0067/22/5/2501/s1.

## Abbreviations

A_2A_AR	adenosine A_2A_ receptor
A_2B_AR	adenosine A_2B_ receptor
ADA	adenosine deaminase
AR	adenosine receptor
BAY60-6583	[2-((6-amino-3,5-dicyano-4-[4-(cyclopropylmethoxy)phenyl]pyridin-2-yl)sulfanyl)acetamide]
BiFC	bimolecular fluorescence complementation
BSA	bovine serum albumin
cAMP	cyclic adenosine monophosphate
CGS-21680	2-[*p*-(2-carboxyethyl)phenylethyl-amino]-5′-*N*-ethylcarboxamido adenosine
DMEM	Dulbecco’s Modified Eagle Medium
DMSO	dimethyl sulfoxide
FCS	fetal calf serum
FPP	farnesyl diphosphate
FTase	farnesyltransferase
G418	geneticin
GAPDH	glyceraldehyde 3-phosphate dehydrogenase
GGPP	geranylgeranyl diphosphate
GGTase-I	geranylgeranyltransferase type-I
GGTase-II	geranylgeranyltransferase type II
GGTase-III	geranylgeranyltransferase type III
GGTI-298	*N*-[4-[2(*R*)-amino-3-mercaptopropyl]amino-2-(1-naphthalenylbenzoyl]-*L*-leucine methyl ester trifluoroacetate salt
GPCR	G protein-coupled receptor
HA	hemagglutinin
HEK	human embryonic kidney
IC_50_	half maximal inhibitory concentration
MSX-2	(3-(3-hydroxypropyl)-7-methyl-8-(*m*-methoxystyryl)-1-propargylxanthine)
NECA	*5′-N*-ethylcarboxamidoadenosine
PBR	polybasic region
PKA	protein kinase A
PS	penicillin-streptomycin
PSB-603	8-(4-(4-(4-chlorophenyl)piperazine-1-sulfonyl)phenyl)-1-propylxanthine
SDS-PAGE	sodium dodecyl sulfate gel electrophoresis
TBS	Tris-buffered saline
WT	wild-type
