# Supplementary material for: Molecular and Pharmacological Characterization of the Interaction between Human Geranylgeranyltransferase Type I and Ras-Related Protein Rap1B
Source: Int J Mol Sci. 2021 Mar 2;22(5):2501. doi: 10.3390/ijms22052501 (PMC7958859; doi:10.3390/ijms22052501)
Supplement: Supplementary file 1 [file ijms-22-02501-s001.pdf]

Supplementary Materials

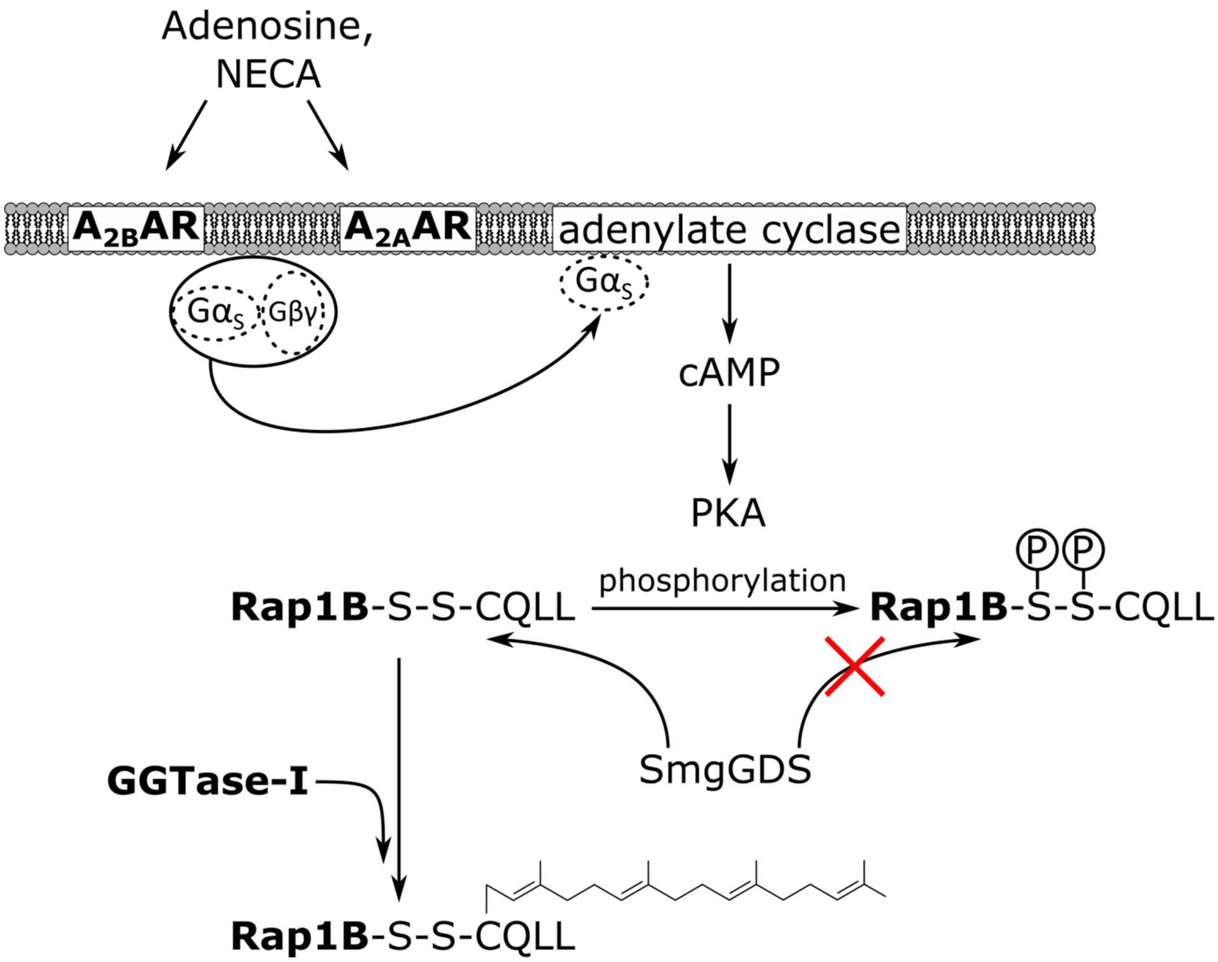

Figure S1. Schematic representation of adenosine receptor signaling influencing Rap1B prenylation. Activation of protein kinase A (PKA) via adenosine receptor signaling results in phosphorylation of the polybasic region (PBR) at the *C*-terminus of Rap1B. This diminishes Rap1B's protein–protein-interaction with SmgGDS and, consequently, its prenylation through GGTase-I. Proteins investigated in this study are shown in bold letters.

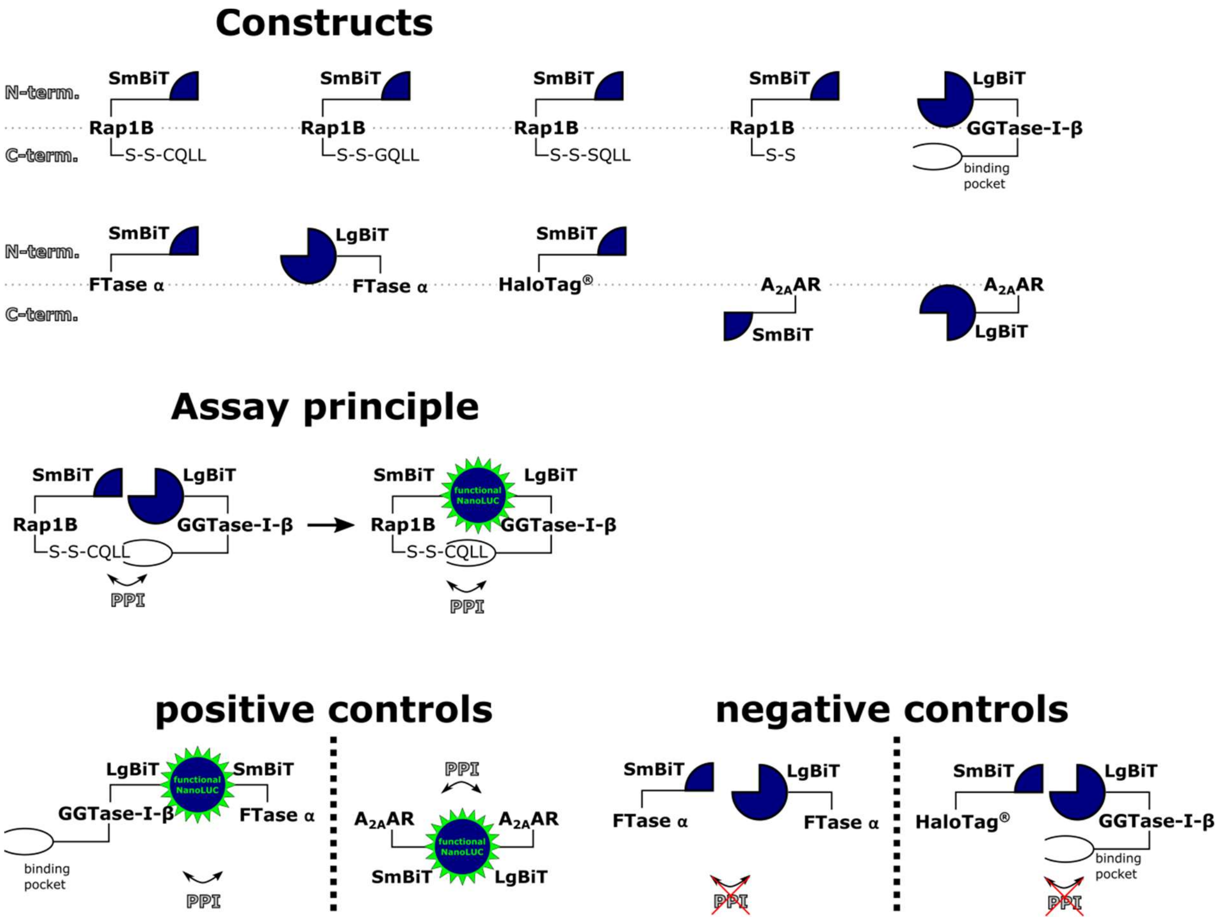

Figure S2. Overview of the applied NanoLuc-fusion-constructs, the assay principle, and the control experiments. The schemes indicate which NanoLuc subunit (SmBiT or LgBiT) is fused to the investigated protein and whether it was fused *N*-terminally or *C*-terminally. Protein–protein interaction (**PPI**) between two proteins fused to SmBiT and LgBiT, respectively, leads to complementation of the NanoLuc enzyme, which in turn results in emission of a light signal.

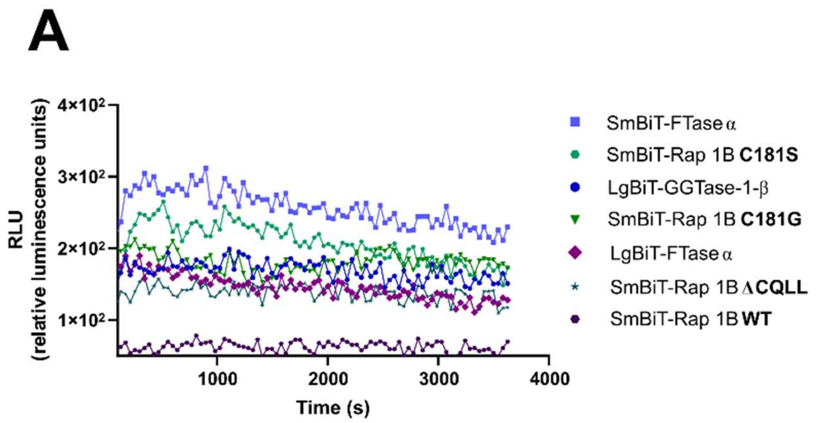

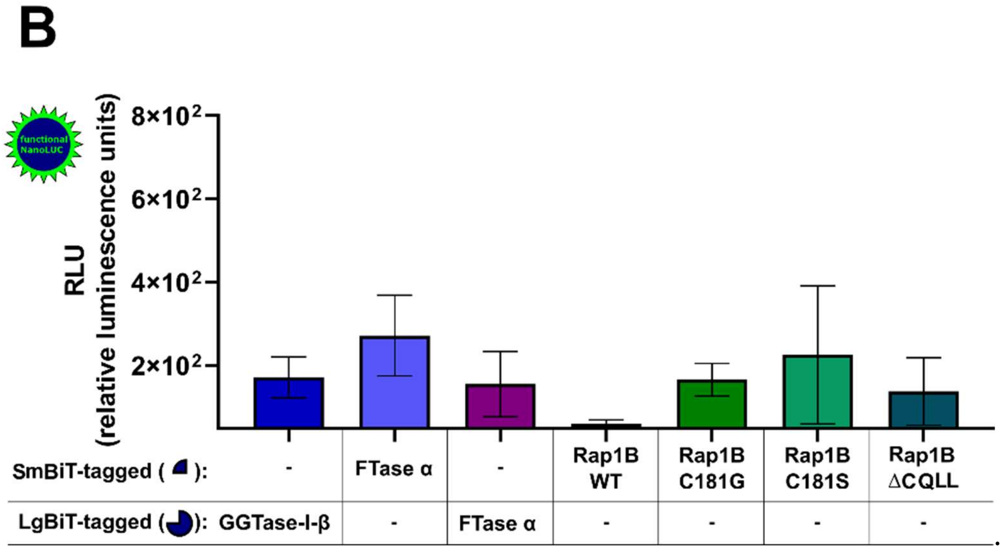

Figure S3. Evaluation of single transfected plasmid constructs for luminescence signals. HEK293 cells stably expressing the A_{2B}AR were transiently transfected with single plasmids encoding LgBiT or SmBiT attached to the *N*-terminus of GGTase-I-β, FTase *α*, Rap1B WT, and Rap1B mutants C181G, C181S, ΔCQLL (each 50 ng DNA/well). **(A)** After 24 h, the NanoGlo live-cell substrate was added and the luminescence was immediately monitored at 25 °C over a time period of 60 min with a luminescence reading taken every 30 s. Points represent the mean of three independent biological experiments performed in single values (*n* = 3). **(B)** After a time period of ~ 1000 s when the luminescence maxima were reached, the relative luminescence units (RLU) were plotted. The data represent the mean RLU ± SEM of three independent biological experiments in single values (*n* = 3).

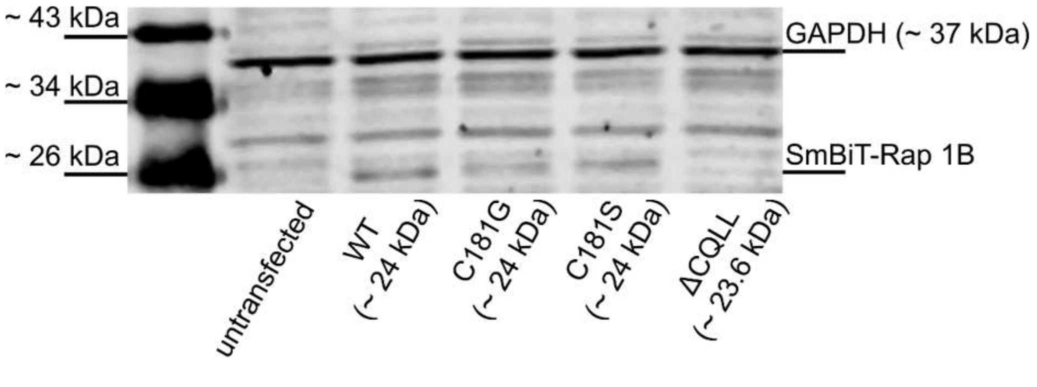

Figure S4. Representative Western blot of SmBiT-Rap1B WT, SmBiT-Rap1B C181G, SmBiT-Rap1B C181S, and SmBiT-Rap1B ΔCQLL expression in HEK293 cells. Glycerinaldehyd-3-phosphat-

dehydrogenase (GAPDH) was used as an endogenous control for protein expression. At least three independent Western blot experiments were performed.

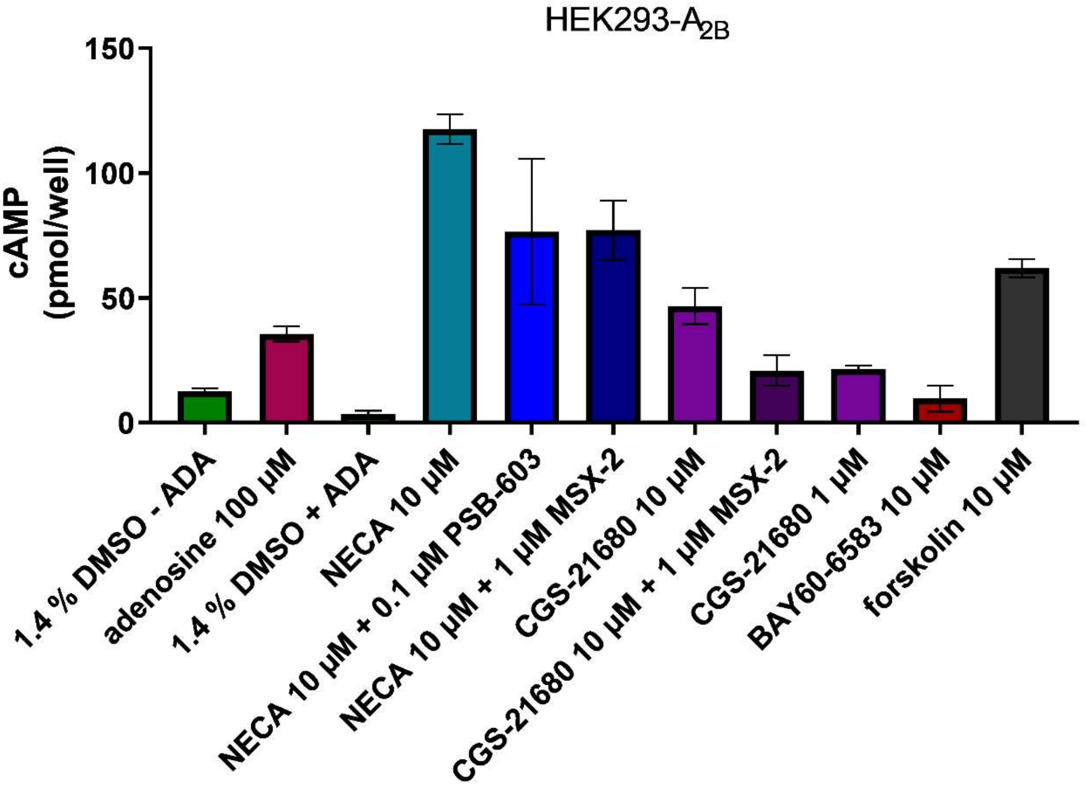

Figure S5. Representative cyclic adenosine monophosphate (cAMP) accumulation experiment at HEK293 cells expressing A_{2B}AR and A_{2A}AR.

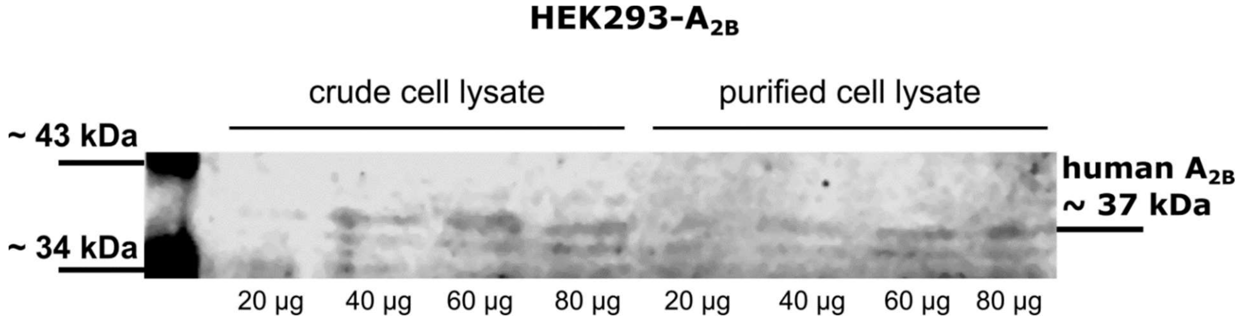

Figure S6. Representative Western blot of A_{2B}AR expression in HEK293-A_{2B} cells.

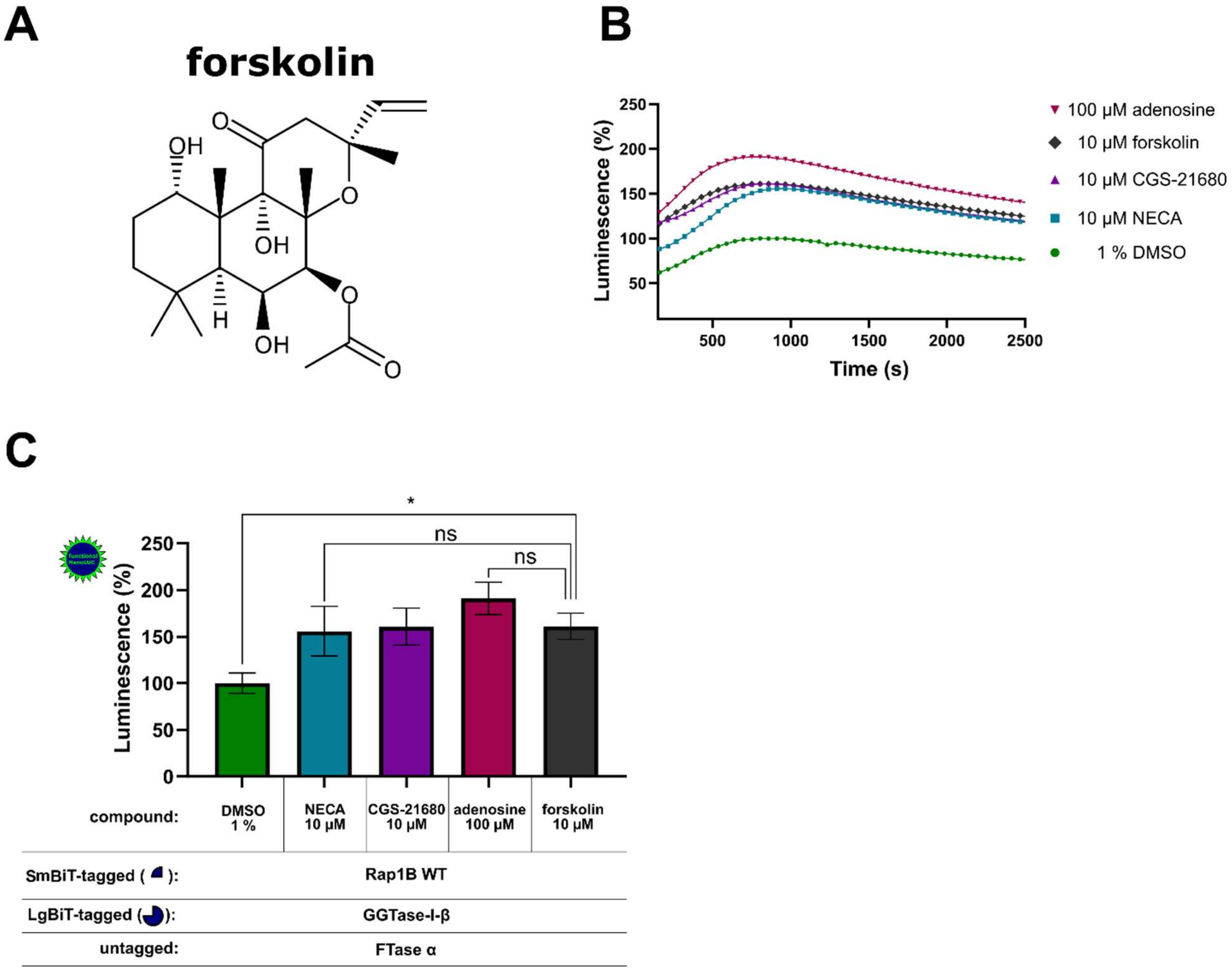

Figure S7. HEK293 cells stably expressing the A_{2B}AR were transiently transfected as described in Figure 1. **(A)** Structure of forskolin. **(B)** Twenty-four hours after transfection, the cells were stimulated for 15 min with the non-selective adenosine receptor agonist NECA (10 μM), the selective A_{2A}AR agonist CGS-21680 (10 μM), the endogenous adenosine receptor agonist adenosine (100 μM), and with forskolin (10 μM). The NanoGlo live-cell substrate was added and the association was immediately monitored at 25 °C over a time period of 42 min, with a luminescence reading taken every 50 s. **(C)** After a time period of ~ 700 s, the maxima of complex formation were reached, and the luminescence was plotted. The luminescence maximum of the untreated LgBiT-GGTase-I-β/FTase α/SmBiT-Rap1B complex (1 % DMSO) was set to 100 %. Significant differences were observed between the untreated LgBiT-GGTase-I-β/FTase α/SmBiT-Rap1B complex (1 % DMSO) and the forskolin (10 μM)-treated Lg-BiT-GGTase-I-β/FTase α/SmBiT-Rap1B complex ($*p < 0.05$). No significant differences were observed between the NECA and adenosine-treated LgBiT-GGTase-I-β/FTase α/SmBiT-Rap1B complex and the forskolin-treated Lg-BiT-GGTase-I-β/FTase α/SmBiT-Rap1B complex (ns, not significant).
